# Using behavior change communication to lead a comprehensive family planning program: the Nigerian Urban Reproductive Health Initiative

**DOI:** 10.9745/GHSP-D-14-00009

**Published:** 2014-12-02

**Authors:** Susan Krenn, Lisa Cobb, Stella Babalola, Mojisola Odeku, Bola Kusemiju

**Affiliations:** a Johns Hopkins Center for Communication Programs, Baltimore, MD, USA; b Johns Hopkins Center for Communication Programs, Nigerian Urban Reproductive Health Initiative, Abuja, Nigeria

## Abstract

**Background:**

The Nigerian Urban Reproductive Health Initiative (NURHI), a 6-year comprehensive family planning program (2009–2015) in 4 cities, intentionally applies communication theories to all program elements, not just the demand generation ones, relying mainly on a theory called ideation—the concept that contraceptive use is influenced by people's beliefs, ideas, and feelings and that changing these ideational factors can change people's behavior.

**Program Description:**

The project used multiple communication channels to foster dialogue about family planning, increase social approval for it, and improve accurate knowledge about contraceptives. Mobile service delivery was started in the third year to improve access to clinical methods in slums.

**Methods:**

Data from representative baseline (2010–11) and midterm (2012) surveys of women of reproductive age in the project cities were analyzed. We also used propensity score matching to create a statistically equivalent control group of women not exposed to project activities, and we examined service delivery data from NURHI-supported clinics (January 2011–May 2013) to determine the contribution of mobile services to total family planning services.

**Results:**

Three years into the initiative, analysis of longitudinal data shows that use of modern contraceptives has increased in each city, varying from 2.3 to 15.5 percentage points, and that the observed increases were predicted by exposure to NURHI activities. Of note is that modern method use increased substantially among the poorest wealth quintiles in project cities, on average, by 8.4 percentage points. The more project activities women were exposed to, the greater their contraceptive use. For example, among women not using a modern method at baseline, contraceptive prevalence among those with no exposure by midterm was 19.1% vs. 43.4% among those with high exposure. Project exposure had a positive dose-response relationship with ideation, as did ideation and contraceptive use. By the end of the observation period, mobile services were contributing nearly 50% of total family planning services provided through NURHI-supported clinics. Propensity score matching found that the increase in contraceptive use in the 4 cities attributable to project exposure was 9.9 percentage points. Intention to use family planning in the next 12 months also increased by 7.5 to 10.2 percentage points across the 4 cities.

**Conclusion:**

Demand-led family planning programs, in which demand generation is the driving force behind the design rather than the conventional, service delivery-oriented approach, may be more suitable in places where expressed demand for contraceptives is low.

## Abstract (precis)

Greater exposure to a comprehensive family planning program in urban Nigeria that emphasized demand generation and communication theory was associated with improved ideation among women (their beliefs, ideas, and feelings about family planning), and more positive ideation was associated with greater contraceptive use, especially among the poor. Improving providers' knowledge, attitudes, and skills was also key. By the end of the observation period, outreach through mobile service delivery contributed nearly one-half of the project clinics' family planning services.

## INTRODUCTION

With a population of 169 million, Nigeria has some of the poorest measures of reproductive health in Africa, including an estimated maternal mortality ratio of 630 deaths per 100,000 live births and an infant mortality rate of 69 per 1,000 live births.^1^ The Government of Nigeria has committed to improving these indicators as part of the Millennium Development Goals (MDGs). For MDG 5 (improve maternal health), a pillar of achievement is increasing the contraceptive prevalence rate (CPR), a core driver of maternal and reproductive health.^2^

The Nigerian Urban Reproductive Health Initiative (NURHI), a comprehensive family planning program encompassing supply, demand, and advocacy interventions, aims to increase voluntary use of contraceptives by 20 percentage points in 4 large Nigerian cities (Abuja, Ibadan, Ilorin, and Kaduna), with the underlying goal of improving the health of Nigerian women and children. The program began in 2009 and is now in its sixth and final year of implementation. It is led by the Johns Hopkins Center for Communication Programs, in partnership with the Association for Reproductive and Family Health and the Center for Communication Programs Nigeria as well as other local organizations for specific implementation needs.

Similar projects are underway or have recently concluded in India (Uttar Pradesh), Kenya, and Senegal. All are funded by the Bill & Melinda Gates Foundation, and all use a similar basic structure built on the documented elements of successful family planning programming,^3^ although context, strategy, and implementation are very different in each country. The 5 objectives common to all the country initiatives are to:

- Integrate family planning into other health services
- Improve the quality of family planning services
- Build private-sector partnerships
- Increase demand for family planning
- Advocate an improved policy environment

NURHI and the other country initiatives are evaluated by an external evaluation project called the Measurement, Learning, & Evaluation (MLE) project. MLE has conducted baseline and midterm surveys to measure the impact of NURHI, and a final evaluation will be available in early 2015. For more information on the initiatives in India, Kenya, and Senegal, see the MLE website (www.urbanreproductivehealth.org), which is designed to share the learning from these programs.

The project's activities, which included performance improvement at facilities, training providers in contraceptive provision, and ensuring efficient and effective commodity logistics systems, will be familiar to anyone who has designed and implemented a comprehensive family planning program; what NURHI has done differently than most programs is to use communication methodologies to adapt each activity—even the service delivery ones—and to put serious and sustained effort and resources into demand generation activities.

NURHI uses communication methodologies to adapt each program activity and places more emphasis on demand generation activities than most other comprehensive family planning programs.

The NURHI initiative was designed based on the hypothesis that *when demand for family planning rises, supply will rise to meet the demand* over time. NURHI defines demand for family planning as the desire and ability among women and/or men to take action to plan their families. Our hypothesis does not imply that one can leave the supply side to itself; it simply reframes the often unstated but very real assumption built into some large-scale family planning programs that *if you build it, they will come.*

Creating demand for family planning was clearly a priority in Nigeria, as just a fraction of women were articulating a desire and need for family planning. For example, in the 2008 Nigeria Demographic and Health Survey,^4^ the national CPR for modern methods was 10.5% (with less than 2 percentage points of growth since the 1999 survey^5^) and 39% of women cited opposition to contraceptive use, but 20% had an unmet need and 21% intended to use contraception in the future. While designing the NURHI project, the Bill & Melinda Gates Foundation shared with program designers an overview of its 2008 reproductive health strategy, in which it estimated through its own calculations that “demand issues comprise 70% of the problem and, therefore, are an even larger driver for achievement of our goals.”

Furthermore, there are adequate sources of short-acting contraceptive methods in parts of the country, including through the nonprofit health sector (the public sector and nongovernmental organizations) and through a robust and entrepreneurial for-profit health care sector that includes patent medicine vendors (who serve as frontline health care providers for a large percentage of Nigerians), pharmacists, and an array of small to large-scale health facilities (general and maternity clinics and hospitals). Interestingly, the majority of family planning users in Nigeria already purchase their contraceptives from the nonclinical private sector.^6^ In NURHI's urban sites, the primary issue was thus not a lack of sites that could provide family planning services, but rather that no one was asking for them. This was supported by the baseline survey, which found that under 1% of women in the 4 intervention cities cited cost, distance, or access as a reason for not using family planning.^6^

The purpose of this article is to describe the activities designed and implemented by NURHI to meet the project's stated objectives and to illustrate how having a demand lens influenced programming decisions in ways that other family planning programs probably would not have considered. We also present findings about the project's outcomes at midterm, primarily from the MLE evaluation surveys.

## INTERVENTION DESIGN AND COMPONENTS

In this section, we introduce the theoretical foundation that underpins the NURHI project and describe in some detail each component of the project, including its formative research, demand generation activities, service delivery interventions, and advocacy activities. The research, strategies, and materials used to design and implement NURHI can be found at www.nurhitoolkit.org.

### Theoretical Foundation

NURHI's overall design and strategy are driven by the project's *theory of change.* NURHI understands the barriers to contraceptive use in its intervention cities to be primarily ones of knowledge, attitudes, and social norms, and the causal pathway to improve the CPR is through changes in these factors at each level of society, from the individual up through communities, service sectors, and the policy environment. Communication is the driver of this change at every level, from demand creation at the individual level, to supportive supervision and training in interpersonal communication at the provider level, to advocacy at the policy level.

**Figure 1.**
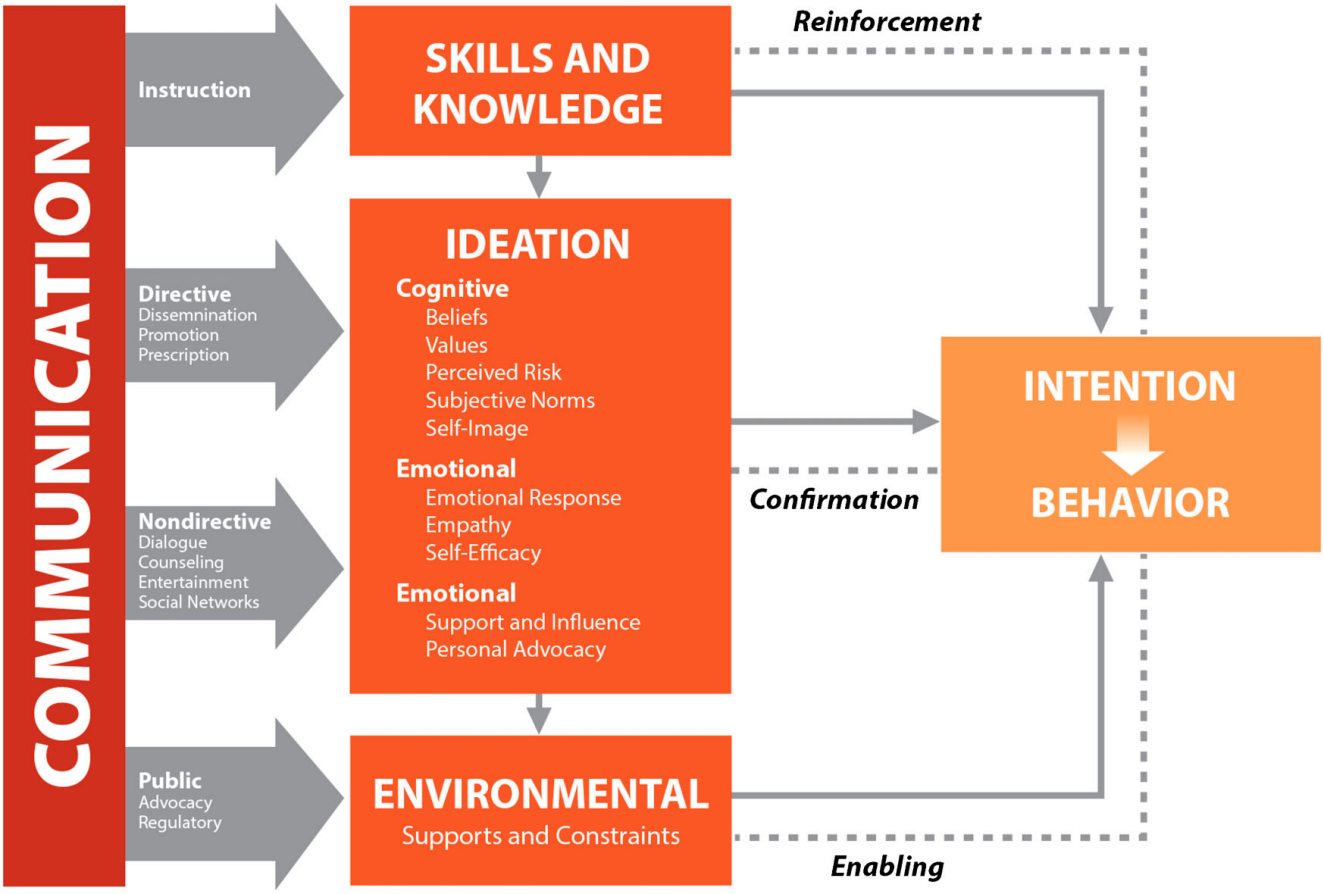
Ideation Model of Communication Source: Health Communication Capacity Collaborative (2014).^8^

In developing strategies for demand generation, service delivery interventions, and advocacy, NURHI has made use of a communication theory called *ideation*. Ideation is the concept that people's actions are influenced strongly by their beliefs, ideas, and feelings (“ideational factors”) and that changing them can change behavior, including contraceptive behavior (Figure 1).^7^ Some of these ideational factors are personal, such as what a person knows about family planning and how they think it will affect them. Others reflect social norms, such as what people believe other people will think of them if they use family planning. The more positive ideational factors a person holds, the greater the likelihood the person will adopt the desired behavior.

The communication theory of ideation holds that people's behaviors are influenced by their beliefs, ideas, and feelings and that changing these ideational factors can change behavior.

While ideation has often been applied to designing demand generation interventions in family planning, we also applied the basic ideas of ideation to designing our service provision activities. We examined service providers' ideas, beliefs, and feelings about family planning, and adapted service delivery interventions to address them. For example, among the “ideas and feelings” that service providers hold is the common belief that women should not use family planning if they only have 1 or 2 children or if they are young, beliefs that pose a barrier to quality family planning provision. Ideation also includes knowledge, which, for a service provider, would encompass skills in clinical care.

### Formative Research

Reflecting our theory of change, we designed the formative research to explore potential barriers to contraceptive use related to knowledge, attitudes, and social norms, and to pay specific attention to ideational factors in both qualitative and quantitative research. The full set of formative research included a household baseline survey with men and women; focus group discussions with men and women (contraceptive users and non-users) and family planning providers of different cadres; a facility assessment survey; and a family planning social mapping survey in 3 of the project cities.

The NURHI project team worked with the MLE evaluation team to tailor the instruments used in the **baseline and midterm surveys** to measure indicators important to this communication-infused program. Questions were tailored to measure ideational factors (partner communication, beliefs and attitudes, correct knowledge, perceptions of peer support, self-efficacy, and perceptions of religious approval), which, taken together, are used as an index predictive of contraceptive use; changing these factors contributes to increased contraceptive use. We also tailored the evaluation to add a baseline survey of men, because although men are not family planning clients for most methods, they are integral to the decision-making process and NURHI needed information about their beliefs, needs, and desires.

**Qualitative research** with users and non-users as well as with service providers was used to complement the baseline survey findings. The research with the providers uncovered and described the biases they held against certain types of clients and methods. Focus groups with men and women explored their beliefs, motivations, fears, and perceptions of use and non-use.

**Figure 2.**
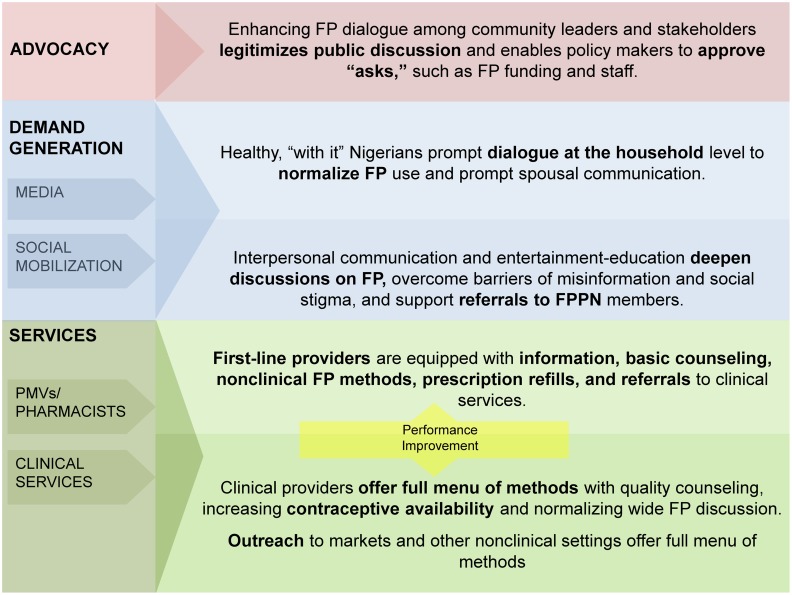
Nigerian Urban Reproductive Health Initiative (NURHI) Interventions Abbreviations: FP, family planning; FPPN, Family Planning Providers Network; PMVs, patent medicine vendors.

A number of important findings emerged from these quantitative and qualitative research methods (Box). Taken together, we concluded that a major barrier to contraceptive use in the project cities was fear and bias. NURHI's interpretation of these data is that people approve of family planning as a concept but believe individual methods are risky. Service providers believe it is their role to uphold social norms around family size, marriage, and spousal consent. These are major challenges, but ones that can be addressed using communication approaches—by encouraging people to talk about family planning and helping to make it a normal part of life, by providing accurate information about the safety of contraceptive methods, and by helping providers use their clinical knowledge, rather than their personal values, in the counseling room. NURHI designed the project's demand generation, service delivery, and advocacy interventions to achieve these goals (Figure 2).

**BOX. Formative Research Findings Guide Program Design**

The baseline survey provided the following essential information that guided the design of program interventions:

- Contraceptive prevalence was low in the 4 project cities, ranging from 19.6% in Kaduna to 33.3% in Ibadan. The majority of modern contraceptive users were using short-acting methods; for example, in Ibadan only 5.4% of married women used 1 of 3 long-acting or permanent methods available (sterilization, IUDs, implants).
- Most women cited no intention to use family planning in the next year (for example, in Ibadan only 7.5% of non-users intended to use contraceptives in the future). The main reasons women gave for not using contraception related to either being pregnant or wanting to be pregnant (36.7% of women in Abuja fit this profile) or having no/infrequent sex (36.2% in Abuja). This indicated to NURHI that a major challenge was to help people think about the benefits to spacing their children and planning their families.
- Fear of specific methods and misconceptions about their side effects was a major non-fertility related reason for not using contraception (in Kaduna, for example, 13.8% of women said they did not use contraceptives due to fear of side effects). The majority of women and men stated that they approved of family planning as a practice, but they held fearful and negative views of actual available methods.
- Despite high levels of awareness of contraceptives (over 90% of women knew of at least 1 method and where to get it), there was limited knowledge of clinical methods (IUDs, implants, and sterilization). Qualitative research showed that what “knowledge” there was of these methods was generally based on myths and misconceptions and contributed to fear of these methods.
- Of women who were using a method, most were using short-acting methods through private-sector pharmacies and drug shops rather than clinical methods from clinics or hospitals with a trained provider. This point dovetails with those above: Women did not know of clinical methods and what they did “know” was negative. Furthermore, women may not have seen access as a barrier because they were not trying to access clinical methods, where services may not be readily available.
- Women and men did not report discussing family planning, contraceptives, or their desired number of children. Spousal discussion is strongly predictive of family planning use,^9^ and so lack of discussion is a barrier.
- While women said religious approval was important, and some felt that their religion did not approve of family planning, a majority of women believed that they could use a contraceptive method despite religious disapproval, a surprising finding. Gender preference was also prevalent but not predictive of non-use, another surprising finding that shaped program interventions.

Qualitative research provided in-depth understanding of the barriers to family planning use:

- Focus groups with service providers showed that the providers had biases and myths and misconceptions about family planning that reflected those of the larger city populations. In particular, service providers believed women should have many children and should not use contraceptives to space them until they have already had a large family. They also disliked providing services to young women, unmarried women, and women with few children. The following quotes from in-depth interviews with service providers illustrate these medical barriers^10^:
  We do not provide family planning to unmarried young girls because it can make them promiscuous.
  —Female, 24 years old, middle income, head of nursing at a private clinic in Kaduna
  I don't like attending to youth because of their involvement in what they are not due for. Also, I don't like attending to the unmarried people.
  —Female, 18–29 years old, owner of patent medicine store in a slum in Ibadan
  Furthermore, sometimes providers perpetuate biases and myths about contraception to potential family planning users. One 21-year-old married woman from Ibadan with 1 child said, “*The advice given to us in the hospital is that the IUD is risky.”*
- A survey of service providers at clinics and hospitals showed that they restricted access to certain methods based on a woman's age, marital status, or parity rather than on medical eligibility. For example, 48% of providers restricted access to injectables if they felt a woman has not had enough children; 60% restricted access to IUDs if a woman is not married; and 30% restricted access to pills without spousal consent.^11^
- Focus groups with women and men found that a major barrier to family planning use was the need for women to obtain their husband's permission to use family planning, but women found it difficult to start a conversation about family planning with their husbands. Both men and women, in general, approved of planning one's family. However, men felt it was the women's responsibility to begin the family planning discussion, and women felt it was the men's responsibility, and so the conversation was not happening.
- Women and men described the need to plan a family as a way to ensure one had only the number of children one could “cater for,” meaning feed, clothe, house, educate, and love. People saw children as a blessing and a gift but also as a great responsibility; they described this responsibility as the reason for supporting family planning. A 24-year-old married woman from Ibadan with 2 children and middle socioeconomic status explained^12^:
  Having too many children is not good. Everyone knows his capacity, and I think it is necessary to limit your childbirth to what your capacity can take you. God will not come down from heaven to help.
- Aside from condoms, people viewed contraceptives as highly medical, requiring medical tests and a perfect fit with one's anatomy. Hormonal and clinical methods were seen as risky—more risky than giving birth to many children. A 30-year-old married man from Ibadan with 1 child and middle socioeconomic status described this fear, which was rooted in misconceptions about contraception^11^:
  I will advise her [his wife] not to do it. Family planning is very dangerous to a person's health. Great caution needs to be exercised.
- Women and men engaged in a social mapping exercise, which enabled the NURHI team to identify community locations that were key points for social interaction that could be used for social mobilization purposes, including markets, places of worship, and schools. It also generated information on commonly used and preferred service delivery points where NURHI could invest in quality improvement, commodity supply, and other service delivery interventions. As this exercise was qualitative and not comprehensive, preferred service delivery sites in NURHI project cities were also identified through the baseline survey.

In urban Nigeria, people approve of family planning as a concept but believe particular methods are risky.

### Demand Generation

NURHI's demand generation strategy for women and men focuses on demedicalizing and demystifying the practice of family planning, including fostering dialogue around family planning—in the home, on the street, at work, in the clinic, in the media; increasing understanding, appreciation, and social approval for planning one's family; improving knowledge and perceptions of family planning methods; and reinforcing existing contraceptive use and reducing discontinuation.^13^

The initial strategy included a 3-phase approach: Phase 1 was designed to increase access to basic family planning information and to heighten awareness of family planning; Phase 2 meant to deepen understanding, discussion, and exploration around the concept of family planning and about specific methods; and Phase 3 was designed to increase the level and localization of communication efforts but was subsequently rolled in with Phase 2 based on timing issues.

From the outset, NURHI's demand side activities have been orchestrated to mutually reinforce one another, in addition to being closely integrated with the service delivery and advocacy objectives. We use multiple communication channels, based on the theory that communication interventions have a synergistic impact, so that hearing or seeing messaging through more than one medium has more impact than hearing or seeing messaging in just one way. Furthermore, the communication activities operate at different levels of the socio-ecological environment, from the individual up through the community and to the policy environment, with messaging designed to address essential cognitive, emotional, and normative ideational factors. The main NURHI demand generation activities consist of mass media, entertainment-education, social mobilization, and integrated branding with a memorable, colorful puzzle logo and tagline that helps tie all program activities together under one identity. The tagline is “Know. Talk. Go.”, meaning “know” your family planning options, “talk” to your partner, and “go” for services.

NURHI employs multiple communication channels to spread the program's messages more effectively.

#### Mass Media

A media campaign featuring the overarching puzzle logo and the “Know. Talk. Go.” tagline uses radio and TV spots and print materials (eg, posters, umbrellas, flyers, t-shirts) to get the word out. Some of the scenes or materials illustrate partner communication; others show barbers or hairdressers discussing family planning with their friends in an open and easy way or couples going to clinics for services, allowing NURHI to model healthy, happy family planning users. For example, in one spot, a couple gets the happy news they are expecting a second child soon after stopping their contraceptive method, refuting the myth of impaired fertility with contraceptive use.

**Figure:**
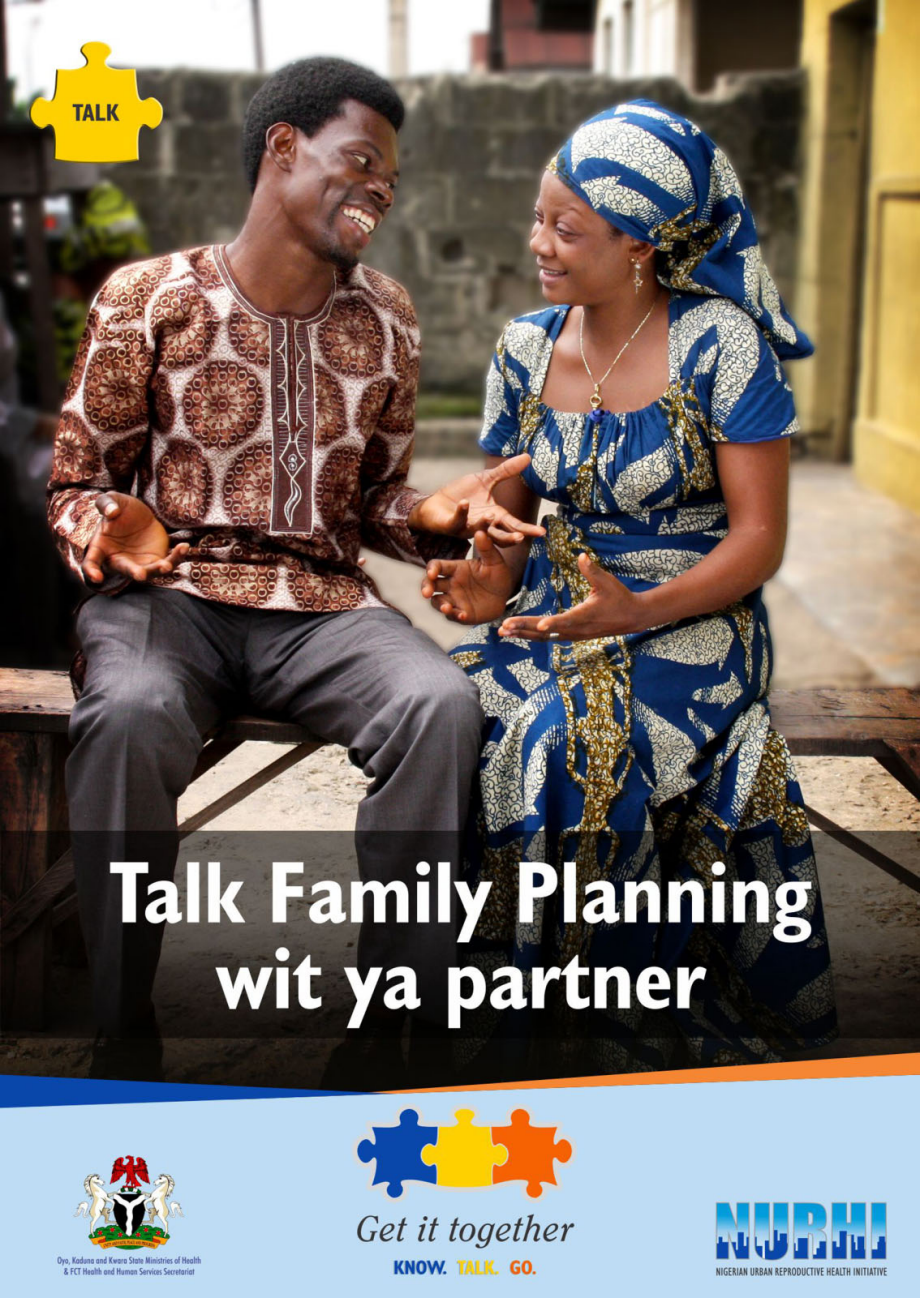
A poster produced by the NURHI project for the “Get It Together” campaign encourages partners to discuss family planning together.

#### Entertainment-Education

A 30-minute weekly radio magazine program (a radio show with various magazine elements, such as listener interviews and call-in “ask the expert” segments) was also produced and broadcast in each project city. These programs include additional content about contraceptive methods, they address myths and misconceptions, and they model discussion of family planning between spouses and with providers. In the initial plans, NURHI had expected to produce one program that could be translated for each location. However, to fully localize it to the specific city context, ultimately a unique program was designed for each site although the format remained consistent. The second phase of the radio programs integrates a live call-in component, with a quiz and an opportunity to ask questions to an “on-air” expert. Radio listening groups, formed within the city environment, are convened on a weekly basis by social mobilizers to listen to and discuss the content of the programs, thus deepening the dialogue, reflection, and understanding of family planning.

#### Social Mobilization

NURHI social mobilizers were chosen not for their expertise in health but for their expertise in talking to people and making connections in their slum communities. In Nigeria, these are hairdressers, barbers, and tailors. Working through professional associations and community-based organizations, NURHI recruited mobilizers from these professions, trained them in family planning, and equipped them with materials, including “Know. Talk. Go.” referral cards. In addition to leading the radio listening groups, they talk to their clientele in their shops about family planning, mobilize clients for family planning outreach services, and discuss family planning at key life events, such as graduations and naming ceremonies. They are now widely sought out by community members, as their participation is considered highly prestigious. This focus in the community has been critical to personalizing the agenda, making family planning a socially acceptable topic, and providing a bridge between the community members and the services. The mobilizers are not paid, which is both a strength and an ongoing challenge for retention and commitment. Recognition of their role and contribution to the well-being of others in the community has inspired many of the mobilizers to continue with the work.

### Service Delivery

NURHI's service delivery component is based on best practices in service integration and quality improvement,^3^^,^^14^ but with the added dimension of treating service providers as an audience for behavior change. The formative research identified key biases among providers related to their attitudes toward family planning, the provision of specific methods, and the women who seek services. Many providers lacked basic family planning knowledge and, in many instances, the technical competency to provide particular services. As NURHI launched and program staff spent time in clinical facilities, it also became apparent that the decrepit family planning facilities (lack of privacy for clients, lack of running water, leaking roofs, dirty floors and walls) were not just an issue of hygiene or safety; they also indicated to providers how little family planning mattered to hospital administrators, which was demotivating to staff.

The issue of **decrepit facilities** is illustrative of how we approached a supply intervention (renovating the facilities) with a demand lens. NURHI viewed the decrepit facilities as an indicator of the ideas and feelings (the ideation) of stakeholders, policy makers, the larger community, and service providers, specifically that they lacked motivation for and did not value family planning. The solution therefore involved advocacy with local stakeholders, engagement with facility administrators, participation of facility staff in the renovation process (coined as the “72-Hour Clinic Makeover”), and a launch of a “new and improved” family planning facility that built support for the providers in their community. It is important to note that the facility renovations generally entailed a coat of fresh paint, scrubbing, connecting a sink to the hospital's water line, and making sure contraceptive commodities and equipment were on hand—not, in most cases, major costs or construction.

“72-Hour Clinic Makeovers” not only improved facility conditions but also engaged facility staff to value family planning.

NURHI also applies a demand lens to improving **counseling sessions between providers and clients**, which we consider to be critical episodes of interpersonal communication. We have ensured that provider training sessions include ample time and priority for interpersonal communication and counseling. In addition, we have made sure that providers have the tools they need to counsel their clients well to provide voluntary, free choice of methods, and we have developed those materials to seamlessly integrate with demand generation outside the clinic walls. For example, counseling materials and job aids were part of the overall NURHI communication approach, with consistent branding, creative approach, and messaging so that both clients and providers would associate what happens inside the clinic with the television, radio, and interpersonal communication they were exposed to in the community.

**Figure:**
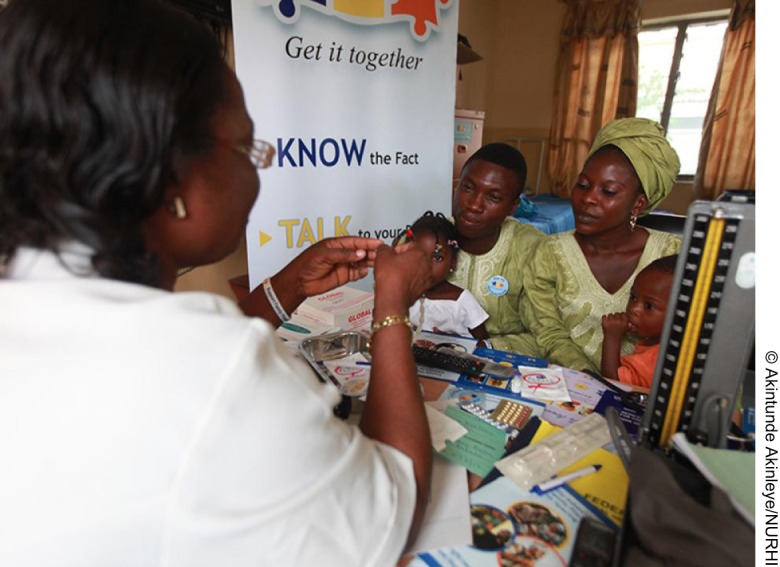
A couple attends a family planning counseling session in Ibadan, Nigeria.

Finally, our **selection of service sites** for the project's clinical interventions was informed by communication approaches by first considering, through the baseline household survey, women's preferences, needs, and behaviors regarding where they access health services. We matched that input with sites with a high volume of clients and also asked women where they spent time in their community, so we had an idea of where we could reach women outside of the clinic. Using this information, we selected service delivery points for clinical quality improvement, almost all of which were public-sector facilities where clinical services were available, plus a broad network of mostly private, nonclinical service delivery sites where providers expressed interest in family planning. We connected all of these providers through a new branded network called the “Family Planning Providers Network” to ensure access to the full basket of services in every project city. We also adopted and adapted a clinical service outreach model to fill a gap in clinical services in slum neighborhoods, in which we provide services on advertised days in tents in markets and in small health posts with no regular family planning providers. These outreach visits are linked to our demand generation work, by using social mobilizers to promote the outreach events and make referrals to them.

Outreach through mobile service delivery provided access to clinical methods in hard-to-reach slum neighborhoods.

### Advocacy

In NURHI's view, policy makers and traditional and religious leaders—as well as service providers—are important audiences in need of communication and “demand generation” just as much as the general public. The difference is in the kind of information they need and in how they can access that information.

Baseline research showed that hearing a religious leader voice support for family planning was an important ideational factor for women and men in Nigeria.^6^ NURHI enlists prominent leaders of multiple faiths to speak publicly and in the media about family planning. The project also developed advocacy kits for each city's policy makers, many of whom are motivated to make progress toward the MDGs, that synthesized baseline data at the city level and highlighted MDG-related trends and how family planning could impact them. In each city, we also formed advocacy groups that included all interested partners working to improve family planning, and these groups oversaw the development and use of the advocacy kits and took ownership for progress.

NURHI enlists prominent faith leaders to speak publicly and in the media about family planning.

While funds for family planning are often allocated in State and Local Government Area budgets, the funds are often not released so that family planning coordinators and facilities can actually use them. Through intensive communication and mentoring, NURHI staff coached government staff in the intricacies of local-level budgeting, requesting processes, and spending decisions, with the result that modest amounts of funding began flowing in many Local Government Areas, where the funds had been previously “stuck.”

## METHODS

Data on the outcomes of the NURHI project come primarily from analysis of the MLE baseline and midterm surveys. The surveys are representative of men and women of reproductive age in each NURHI project city. The same women were interviewed for the baseline and midterm surveys, providing a unique longitudinal sample in which sophisticated analytical techniques could be applied to have greater confidence that results of the project could be attributed to exposure to the interventions.^15^ (The men's survey was cross-sectional, however.) The baseline survey of women and men was conducted in 2010–2011 and the midterm survey of women in 2012.^6^^,^^16^

We also conducted additional analysis of the MLE data using a technique called propensity score matching (PSM). This technique allowed us to estimate the probability (propensity) that a woman will be exposed to the program activities and to create an unexposed control group that is statistically equivalent to those exposed. Using PSM, we estimated what the CPR *would have been* among the women exposed to the NURHI project had they not been exposed to it. The difference between the CPR of the women exposed to the NURHI project and the estimated CPR of those same women had they not been exposed is considered the treatment effect of the intervention.

Finally, we performed secondary analysis of the MLE data to determine whether there was a positive relationship between communication activities and ideation (factors such as beliefs, spousal discussion, perceived peer behavior, perceived self-efficacy, and personal advocacy), and whether there was a positive relationship between ideation and contraceptive use. Specifically, 32 ideational items were measured across 3 domains: cognitive, emotional, and social interaction. The items included aspects of contraceptive awareness (12 items), myths and rumors about contraceptives (8 items), perceived self-efficacy to take action regarding contraceptive use (7 items), and approval of leaders talking about family planning (2 items), as well as descriptive norms about contraceptive use in one's community, personal advocacy for family planning, and perceived social support for personal use of contraceptives. The resulting scores were then categorized into quintiles denoting women's overall level of ideation: very low (8 items or fewer), low (9–10 items), medium (11–12 items), high (13–15 items), or very high (16 or more items).

Women's ideation scores were based on 32 variables across cognitive, emotional, and social interaction domains.

In addition to exploring the effect of demand generation activities on contraceptive use, we also examined service delivery data from NURHI-supported clinics between January 2011 through May 2013 to determine the proportion of clinic-provided family planning services attributed to outreach visits.

## RESULTS

### Program Exposure

The MLE midterm survey measured people's exposure to various NURHI messages and strategies and computed overall exposure to the NURHI program by summing up people's exposure to multiple items. Overall program exposure is presented in 4 categories:

1. No exposure
2. Low (knew of 1 or 2 NURHI activities)
3. Medium (knew 3–6 activities)
4. High (knew 7 or more activities)

About 80% of women in the 4 project cities reported some exposure to the NURHI project: 24% reported low exposure, 32% medium, and 25% high, with the remaining 19% reporting no exposure.^16^

### Myths and Misconceptions

Between baseline and midterm, the percentage of women who believed in myths or had misconceptions about contraception declined. For example, the percentage of women who believed incorrectly that “contraceptives are dangerous to your health” dropped by 17 percentage points in Ilorin, from 37.4% to 20.4 %, and by about 15 percentage points in Ibadan, from 57.1% to 42.2%.^16^ Similarly, the percentage who believed that “contraceptives can harm your womb” decreased by 15.7 percentage points in Ilorin, from 33.6% to 17.9%; by 12.5 percentage points in Ibadan, from 49.8% to 37.3%; and by 9 percentage points in Abuja, from 33.4% to 24.1%.^16^

### Intention to Use Contraception

**Figure 3.**
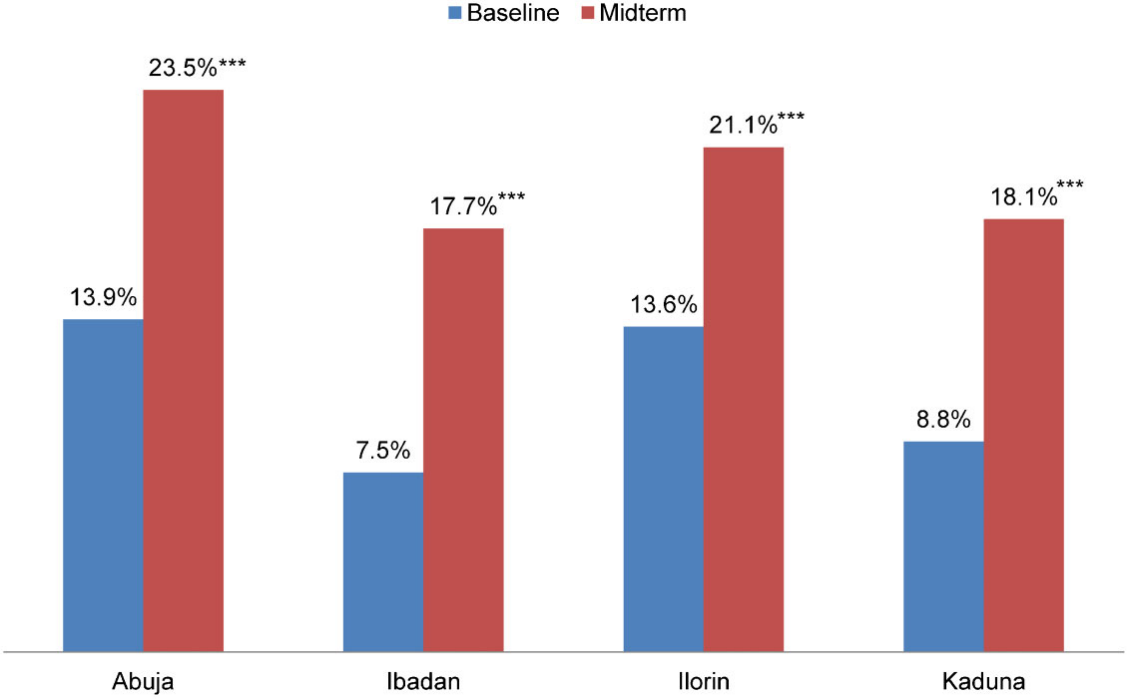
Percentage of Women Not Currently Using Contraception Who Intend to Use a Method in the Next 12 Months at Baseline (2010/11) and Midterm (2012), by NURHI Project City *** *P* < .001.

In each project city, there was a significant upward trend in the percentage of women intending to use contraception. For example, in Abuja and Ibadan, the percentage of women who intended to use contraception in the next 12 months increased significantly by 10 percentage points in each city, from 13.9% to 23.5% in Abuja and from 7.5% to 17.7% in Ibadan (Figure 3). In Ilorin and Kaduna, intention to use increased significantly by nearly 8 percentage points in each city.

Intention to use contraception in the future increased in each project city.

### Contraceptive Use at Baseline and Midterm

Between baseline and midterm, use of modern methods among married women increased in each city, although the change varied widely between the 4 cities (Table 1). For example, in Abuja, 31.9% of married women were using modern contraception at baseline; the percentage increased slightly at midterm to 34.2%, but the change was not statistically significant. On the other hand, in Kaduna, the modern CPR increased by 15.5 percentage points between baseline and midterm, from 19.6% to 35.1% (*P* < .001).

**Table 1.** Modern Contraceptive Prevalence Rate Among Married Women, at Baseline and Midterm, by NURHI Project City

**City**	**Baseline**	**Midterm**	**Percentage Point Change**
Abuja	31.9%	34.2%	+2.3
Ibadan	33.3%	36.9%	+3.6*
Ilorin	26.9%	34.9%	+8.0***
Kaduna	19.6%	35.1%	+15.5***

* *P* < .05; *** *P* < .001.

One factor in those differences is the difference in the modern CPR at baseline between the cities. In Kaduna, for example, the low level of contraceptive use (19.6%) at the start of the project may have represented pent-up need for access to family planning services, resulting in the notable improvement at midterm. The cities differ demographically, politically, culturally, and religiously, and these factors may also have contributed to the different results in each city. It is interesting to note, however, that the modern CPR in the 4 cities at midterm is similar (between 34.2% and 36.9%), whereas the rates were more variable at baseline (between 19.6% and 33.3%). In addition, of note is that the modern CPR increased substantially among the poorest wealth quintiles in NURHI project cities, on average, by 8.4 percentage points.^17^

### Contraceptive Use by Level of Exposure to the NURHI Program

Longitudinal data from the MLE baseline and midterm surveys show that (reported) exposure to several of the NURHI communication interventions was significantly associated with higher levels of contraceptive use. The greatest effects were associated with exposure to the local-language radio entertainment-education programs, social mobilization activities, and television spots.^15^

**Figure:**
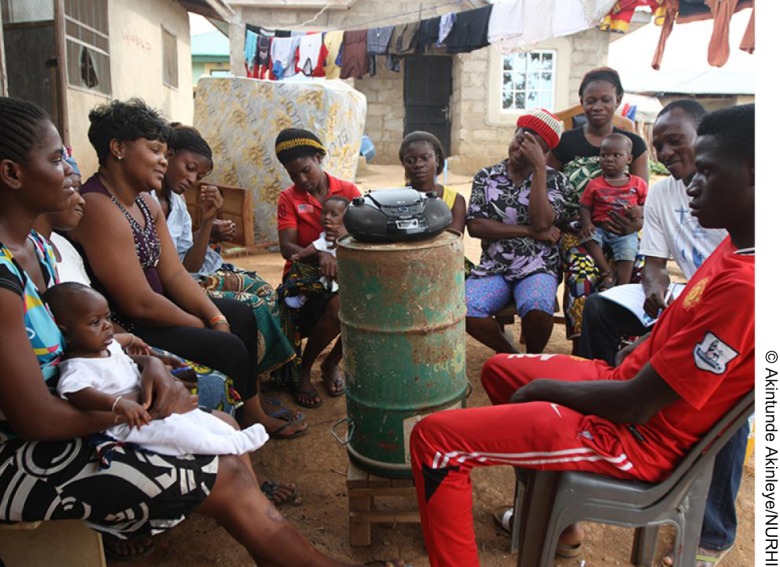
Members of a radio listeners' club listen to and discuss the family planning radio magazine and drama, *Second Chance*, produced by NURHI.

**Figure 4.**
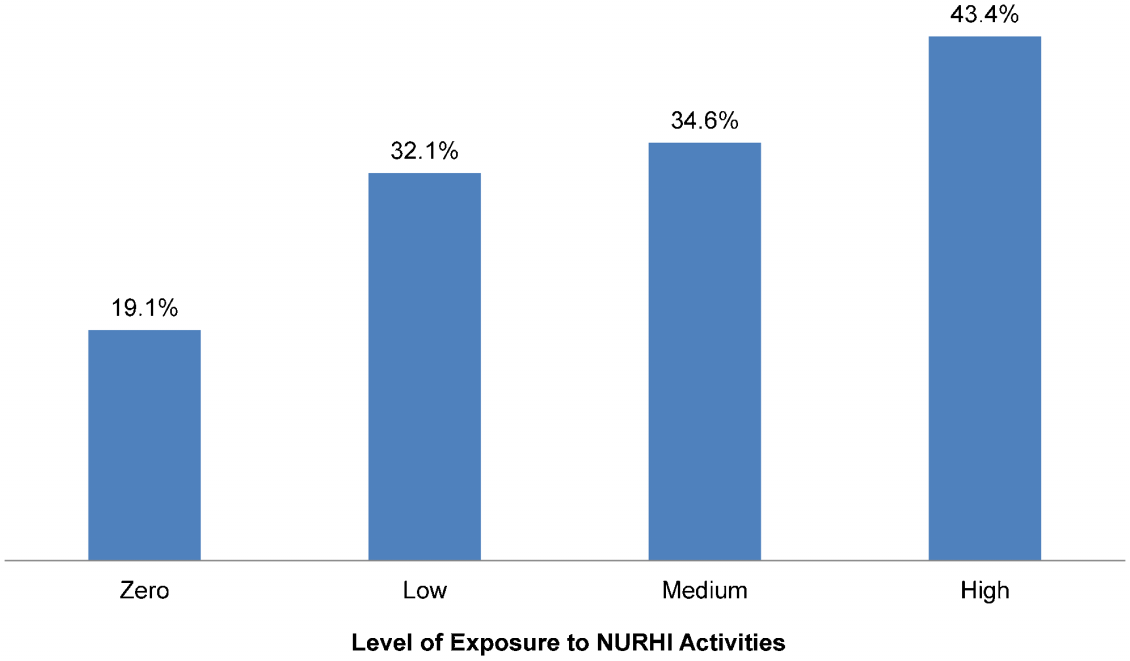
Contraceptive Prevalence at Midterm Among Married Women Who Were Not Using a Modern Method at Baseline, by Level of Exposure to NURHI Activities, N = 1,992 Significance of differences across groups: *P* < .001.

Analysis of CPR data by women's reported level of exposure to NURHI project activities shows that, among married women not using a modern method at baseline, 19.1% were using contraception at midterm among those reporting no exposure to NURHI activities compared with 32.1% among those with low exposure (Figure 4). Contraceptive prevalence increased positively and linearly with greater exposure (medium exposure, 34.6%; high exposure, 43.4%).

Contraceptive prevalence increased positively and linearly with greater exposure to NURHI activities.

We used propensity score matching to better understand whether changes in behavior (contraceptive use) were attributable to exposure to NURHI's demand generation activities. This analysis showed that the CPR among the matched control group would have been 25.9% had the women not been exposed to the NURHI program, compared with the actual (observed) CPR of 35.8%. These data suggest that the increase in contraceptive use (ie, the treatment effect) attributed to exposure to the program was 9.9 percentage points.

### Ideational Factors and Contraceptive Use

Analysis of longitudinal data from the baseline and midterm surveys also finds that 9 of 10 measured ideational factors increased significantly. For instance, the percentage of women who perceived there was peer support for family planning increased significantly from 22.8% to 42.4% (*P* < .001) between baseline and midterm (Table 2). Similarly, the percentage of women who had positive attitudes toward family planning rose from 53.7% to 70.9% (*P* < .001).

**Table 2.** Ten Ideation Factors at Baseline and Midterm That Predict Contraceptive Use

**Ideation Factor**	**Description**	**Baseline**	**Midterm**	**Significance of Change**
Contraceptive methods knowledge	Percent of married or cohabiting women with knowledge of at least 3 modern methods	55.5%	69.2%	*P* < .001
Beliefs/attitudes about family planning	Percent of married or cohabiting women with highly positive attitudes toward family planning	53.7%	70.9%	*P* < .001
Attitudes toward government officials talking about family planning	Percent of married or cohabiting women who approved of government officials speaking publicly about family planning	83.0%	91.4%	*P* < .001
Attitudes toward religious officials talking about family planning	Percent of married or cohabiting women who approved of religious leaders speaking publicly about family planning	58.6%	72.2%	*P* < .001
Spousal communication	Percent of married or cohabiting women who discussed the number of children with spouse during the last 6 months	29.8%	30.8%	Not significant
	Percent of married or cohabiting women who needed spousal permission to use family planning	75.4%	77.4%	Not significant
Perceived peer behavior	Percent of married or cohabiting women with most friends using a modern contraceptive method	8.2%	17.6%	*P* < .001
Perceived self-efficacy	Mean score for perceived self-efficacy to take relevant actions in favor of contraceptive use (range, 0–6)	3.1	3.6	*P* < .001
Family size preferences	Percent of married or cohabiting women who indicated wanting families of 3 or fewer children	14.7%	17.4%	*P* < .05
Perceived peer support	Percent of married or cohabiting women who perceived peer support for family planning	22.8%	42.4%	*P* < .001
Personal advocacy	Percent of married or cohabiting women who encouraged friends to go for family planning services	17.1%	24.2%	*P* < .001

**Figure 5.**
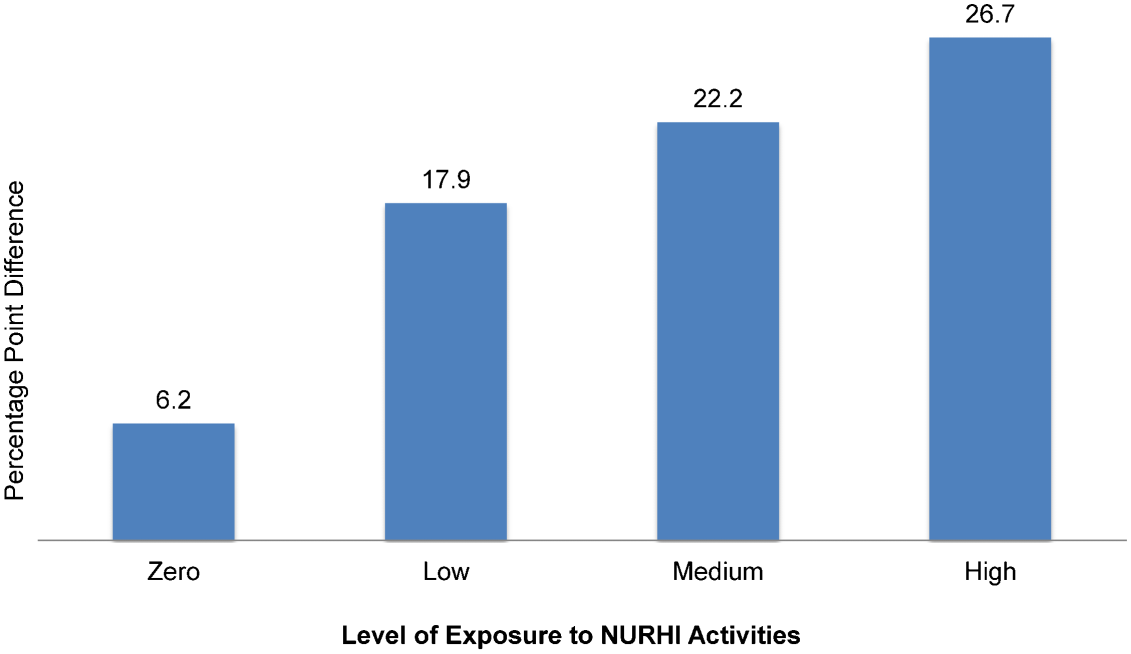
Change in Perceived Peer Support for Family Planning Between Baseline and Midterm, By Level of Exposure to NURHI Activities, N = 4,331 Significance of change in perceived peer support is *P* < .05 for zero exposure and *P* < .0001 for low, medium, and high levels of exposure.

The data also show that level of exposure to program activities had a positive dose-response relationship with these ideational factors. For example, the percentage of women who perceived there was peer support for family planning increased by 6.2 percentage points among women reporting no program exposure, and the percentage increased significantly and linearly with each level of exposure: from a 17.9 percentage point increase among women with low exposure to a 26.7 percentage point increase among women with high exposure (Figure 5).

**Figure 6.**
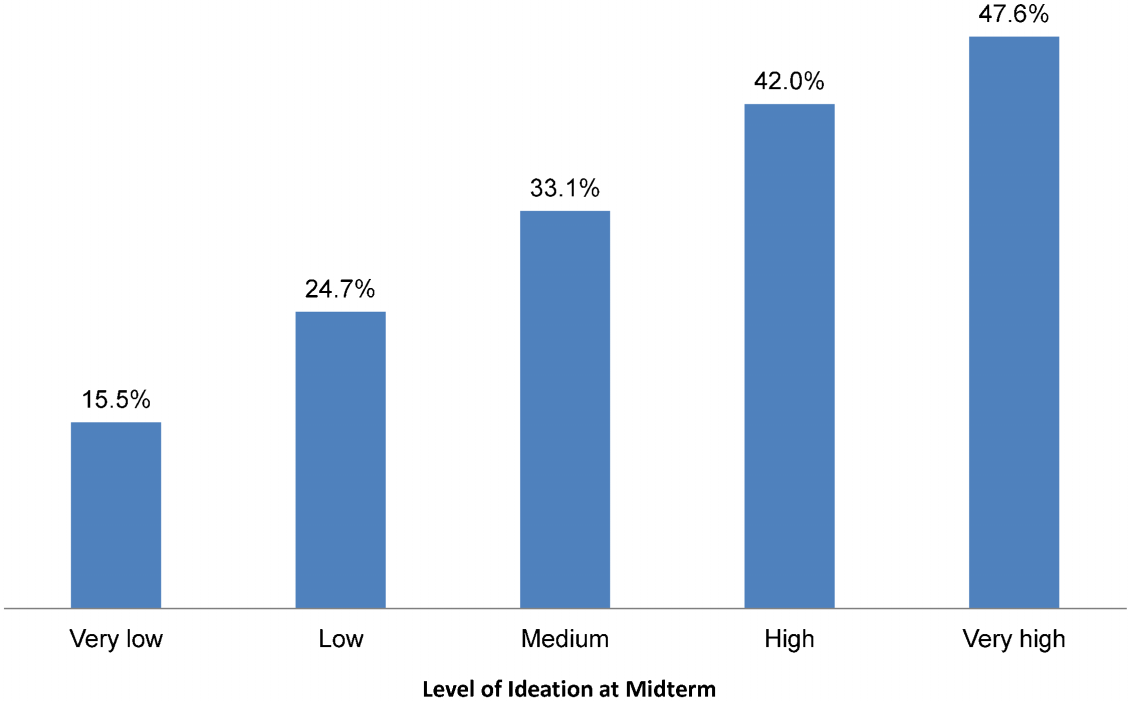
Contraceptive Prevalence at Midterm Among Married Women Who Were Not Using a Modern Method at Baseline, by Level of Ideation at Midterm, N = 1,992 Significance of differences across groups: *P* < .001.

Furthermore, analysis of CPR data by women's level of ideation shows that the more positive ideational factors that women had, the greater their contraceptive use. Among women not using a modern method at baseline, 15.9% of those with very low ideation at midterm were using contraception compared with 28.2% of those with medium ideation and 47.3% of those with very high ideation (Figure 6).

The more positive ideational factors that women had, the greater their contraceptive use.

### Contribution of Clinical Outreach

**Figure 7.**
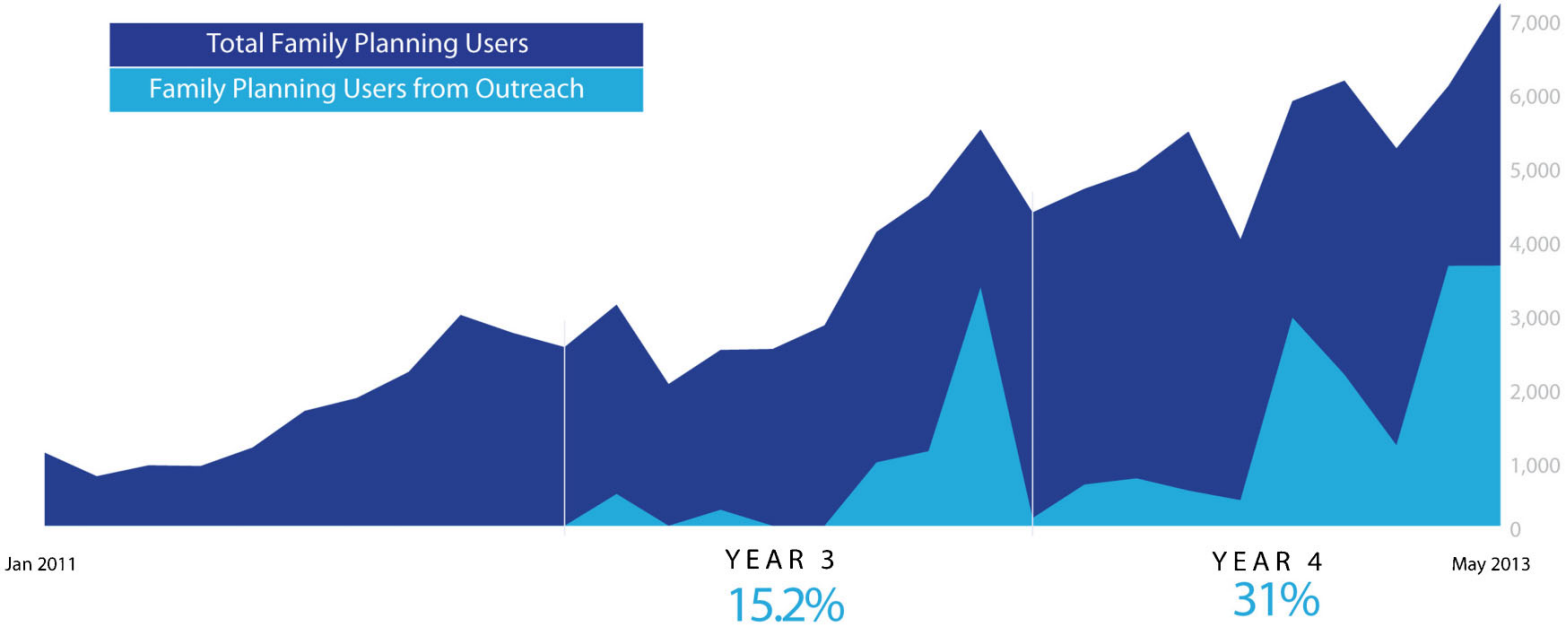
Family Planning Users Served by NURHI-Supported Clinics and Through Associated Outreach Visits, January 2011–May 2013 On average, outreach visits contributed, in the third year of the project, 15.2% of total family planning services provided by NURHI-supported clinics and 31% in the fourth project year.

Figure 7 shows the contribution of clinical outreach to the total number of clients served by the high-volume sites where NURHI has trained and supported providers. Between 2009 and 2011, the NURHI project worked with selected public-sector sites to improve their facilities and quality of services. In the third year of the project (2012), NURHI began dispatching family planning outreach staff to hard-to-reach slum areas. Between January 2011 and May 2013, the number of family planning users served by NURHI-supported facilities steadily increased, from about 1,000 users per month to about 7,000 total users in May 2013 (Figure 7). In 2012, when NURHI started conducting outreach visits, the outreach visits contributed, on average, about 15% of these total family planning users, and the share increased to 31% in the fourth year of the project. At the end of the observation period, outreach visits were contributing nearly half of total clinical services supported by NURHI. Note that the full number of contraceptive users is better represented by data from the midterm survey since women access many sources for family planning, including private-sector providers such as pharmacists and drug shop owners.

By May 2013, outreach visits were contributing nearly half of total clinic family planning services supported by NURHI.

## DISCUSSION

While changes to the contraceptive prevalence rate in the cities where NURHI works have been variable, sophisticated analysis of the longitudinal data indicate that NURHI's demand generation activities are indeed significantly associated with increased contraceptive use in the cities. In particular, the data support the theoretical foundation on which NURHI was based—that is, the communication theory that holds that changing ideational factors, such as knowledge, attitudes, and beliefs, increases the chances of changing people's behavior. Women's use of contraception at midterm increased linearly with increasing levels of ideation. Similar findings have been reported in Bangladesh, Burkina Faso, and the Philippines.^18^^–^^20^ Ideational factors can be thought of as *positive* risk factors, similar to how certain behaviors are risk factors for disease. For example, just as obesity, diet, exercise, and genetics are all risk factors for heart disease, ideational factors are “risk factors” for the positive behavior of family planning. And just as with risk factors for heart disease, the more factors a person has, the more likely that person is to have the outcome, in this case, contraceptive use. When designing family planning programs, this means that program planners can consider the entire scope of ideational factors that are predictive of contraceptive use and select a group of factors to target that are: (1) currently not prevalent, so there is room for growth, and (2) amenable to change. Ideal family size, for example, is an important ideational factor, but it may not always be feasible for programs to address for both practical and political reasons.

Data from the NURHI project also demonstrate that using a combination of communication channels, such as mass media, interpersonal, and communication channels, enhances the effect of communication interventions. Exposure to more NURHI communication activities was associated both with higher levels of ideation among women and with higher levels of contraceptive use. In this way, communication works like a drug, with a dose-response effect.^21^ As the “dose” (number of communication activities to which a person is exposed) increases, so does the impact on ideation and contraceptive use. It is not simply that more exposure to the same communication increases response, but that exposure to multiple channels of communication increases response. This is one reason why NURHI was designed with television, radio, social mobilization, and clinic-based communication interventions—to maximize the types of interventions people experience. Another reason to use multiple communication channels is to maximize the chance of exposure in general, as no one channel reaches everyone. In addition, different channels have different uses, for example, radio is useful for modeling change through entertainment-education while interpersonal communication helps to deepen knowledge; together, the messages communicated through multiple channels become mutually reinforcing.

Intention to use contraceptives is an important indicator for NURHI, because it gives an indication of likely future users—women who might not be ready to use contraception now due to pregnancy or other factors but who want to plan their families. In each NURHI project city, there was an upward trend in the percentage of women saying they intended to use contraception in the next 12 months. In an analysis of data from 27 Demographic and Health Surveys conducted between 1993 and 1996, for each 1% increase in intention to use contraception, there was nearly a 1% rise in contraceptive adoption.^22^

Clinical outreach through mobile services played an important role in improving contraceptive use. Women responded enthusiastically to having family planning services brought to their own neighborhoods. By the end of the observation period, nearly one-half of the services provided by NURHI-supported public-sector clinics came from these outreach visits. Mobile service delivery has shown great success in other projects.^23^ In the urban slums supported by NURHI, women do, technically, have access to family planning facilities within a reasonable distance. But NURHI's mobile services put the convenience and needs of family planning users first, by having service providers travel to them, rather than expecting women to travel to the service provider.

Many large-scale family planning programs tend to be “service-led,” that is, informed by a service-delivery and health systems strengthening approach. Such a program would typically be managed by a partner known for its service delivery expertise and budgeted with service delivery absorbing the majority of program funds, with service delivery needs setting the tone and pace of the project. If supported adequately by demand generation and other high-impact practices,^6^ the service-led approach may be the appropriate design for a given context. However, given that demand generation and communication interventions have been shown to increase family planning use in Nigeria and elsewhere,^24^^–^^26^ it is worth exploring whether an alternative project strategy is effective.

An alternative strategy is for a “demand-led” program, such as that of the NURHI project, which was designed with demand generation as its driving force. What does it mean for a family planning project to be demand-led rather than service-led? It means that from the outset of design, program planners put potential and current family planning users at the forefront, along with their barriers and challenges to using family planning and their desires and hopes. With that insight as a starting point, the demand-led project would *then* design the appropriate systems, supplies, provider inputs, and communication interventions needed to serve the potential and current users. While using this approach, NURHI has come to see the locus of the program as the space between husband and wife, or between romantic partners, rather than at the clinic. The catalyst happens in the home; the rest of the (very substantial) work involves making sure the couple is supported and enabled, both in the community and in the clinic, to plan their family.

A “demand-led” family planning program puts potential and current family planning users at the forefront and uses demand generation as its driving force.

We cannot yet assert whether the demand-led approach is effecting the CPR *faster* than the standard approach, but we can say that it is working. The NURHI hypothesis is that at some point, when CPR has reached a high enough level, family planning will become an ordinary part of family life, and people will feel that their community supports it to such an extent that demand for family planning will be self-maintaining. It is at that point that demand will truly drive supply, leading to sustained demand with providers working to meet it. That does not mean that no further investment will be needed when this occurs; health systems must be funded. But NURHI does believe that an investment in making family planning a social norm—whereby women perceive contraceptive use is ubiquitous, approved, and supported by family, community, and influential leaders—will lead, in time, to a level of demand that will prevent the CPR from falling back down to the low levels now common in some countries such as Nigeria. We have not gotten there yet, but we are headed in the right direction.

Investing in making family planning a social norm will lead, in time, to self-sustaining levels of demand for contraception.

## Notes

**Peer Reviewed**
